# Effect of an Ergonomics-Based Educational Intervention Based on Transtheoretical Model in Adopting Correct Body Posture Among Operating Room Nurses

**DOI:** 10.5539/gjhs.v8n7p26

**Published:** 2015-11-03

**Authors:** Zeinab Moazzami, Tahere Dehdari, Mohammad Hosein Taghdisi, Alireza Soltanian

**Affiliations:** 1 School of Health, Tehran University of Medical Sciences, Tehran, Iran; 2 School of Health, Iran University of Medical Sciences, Tehran, Iran; 3 School of Health, Hamadan University of Medical Sciences, Hamadan, Iran

**Keywords:** body posture, ergonomics, nurse, operating room, transtheoretical model

## Abstract

**Background:**

One of the preventive strategies for chronic low back pain among operating room nurses is instructing proper body mechanics and postural behavior, for which the use of the Transtheoretical Model (TTM) has been recommended.

**Methods:**

Eighty two nurses who were in the contemplation and preparation stages for adopting correct body posture were randomly selected (control group = 40, intervention group = 42). TTM variables and body posture were measured at baseline and again after 1 and 6 months after the intervention. A four-week ergonomics educational intervention based on TTM variables was designed and conducted for the nurses in the intervention group.

**Results:**

Following the intervention, a higher proportion of nurses in the intervention group moved into the action stage (*p* < 0.05). Mean scores of self-efficacy, pros, experimental processes and correct body posture were also significantly higher in the intervention group (*p* < 0.05). No significant differences were found in the cons and behavioral processes, except for self-liberation, between the two groups (*p* > 0.05) after the intervention.

**Conclusions:**

The TTM provides a suitable framework for developing stage-based ergonomics interventions for postural behavior.

## 1. Introduction

Work-related musculoskeletal disorders (WMSD), especially lower back pain are common among operating room nurses (Sheikhzadeh, Gore, Zuckerman, & Nordin, 2009). Awkward posture, prolonged standing and fixed postures, holding and managing equipment such as retractors during surgical procedures and pulling/pushing or lifting patients, heavy instruments and equipments sets are the major contributing factors in WMSD development in this group (Meijsen & Knibbe, 2007).

One of the preventive strategies for chronic low back pain is instructing proper body mechanics and posture (Owen, Keene & Olson, 2002). Studies showed that ergonomics training for maintaining an adequate body posture while at work reduces the prevalence of WMSD among nurses (Sheikhzadeh et al., 2009; Owen et al., 2002).

It is worth mentioning that individuals are in different stages of readiness to adopt the appropriate body posture, any educational approach should identify these differences and accordingly design and provide interventions tailored to an individual’s current stage of change (Keller, Herda, Ridder, & Basler, 2001).

The Transtheoretical Model (TTM), one of the stage models, postulates that people differ in their readiness to adopt a new behavior. According to the TTM, individuals adopting a new behavior go through six stages of change: (a) pre-contemplation - no intention of changing behavior within the next 6 months; (b) contemplation-intends to make a change behavior within the next 6 months; (c) preparation - intends to make a change behavior within the next 30 days and have a plan of action; (d) action - have made overt behavior change within the past 6 months; (e) maintenance - have maintained the overt change in behavior for over six months and (f) relapse - returning to older behavior (Adams & White, 2005). TTM assumes that progressive movement from stage to stage is also linked to differences in perceived self-efficacy (refers to the degree of confidence an individual has about his/her ability to perform a specific behavior), decisional balance (reflects the weighting of the perceived pros (advantages) and cons (costs or barriers) of behavior change). The processes of change are covert and overt activities people use to advance through their stages of change; the ten processes include five experimental and five behavioral processes; the five experimental processes include awareness-raising, dramatic relief, environmental re-evaluation, self-reevaluation and social liberation. The five behavioral processes consist of counter conditioning, stimulus control, helping relations, self-liberation and reinforcement management (Patten, Vollman, & Thurston, 2000; Prochaska, Di Clemente, & Norcross, 1992; Rodgers, Courneya, & Bayduza, 2001; Prochaska, Velicer, DiClemente, & Fava, 1988; Sharma & Romas, 2008; Grimley, Prochaska, & Prochaska, 1997). Although, use of TTM has been recommended for postural behavior (Keller et al., 2001), few studies have used this model for developing ergonomics-based educational interventions (T. Mohammadi Zeidi, Morshedi, & B. Mohammadi Zeidi, 2011). Given the importance of ergonomics training in adopting good body posture for nurses in the operating room environment (Owen et al., 2002) and the clear need for developing stage-based interventions in this field, the present study was designed to determine the effect of a TTM-based ergonomics educational intervention in adopting correct body posture among operating room nurses working in hospitals.

## 2. Material and Methods

### 2.1 Design and Samples

This Quasi-randomized controlled trial study was conducted from April 2011 to July 2012. A convenience sample of 84 nurses in the contemplation and preparation stages in terms of body posture with more than 6 months of job experience in the operating room and without any history of chronic lower back pain or previous lower back surgery were randomly chosen from the four hospitals of Hamadan (21 nurses from each hospital). Nurses from two hospitals were assigned into the intervention group, while those from the two remaining hospitals were assigned to the control group. Two nurses in the control group refused to take part in the study and finally, 42 nurses participated in the intervention group and 40 in the control group. The study was approved by the ethics committee of Tehran University of Medical Sciences and written informed consent was obtained from all the nurses.

### 2.2 Study Instrument and Measures

Demographic characteristics and TTM items were measured by a self-administered questionnaire, the completion of which took 20 to 25 minutes. Work-related posture for the prevention of lower back pain in the operating room was measured using the observational checklist that was completed by observers before the intervention. To develop the TTM items, we reviewed the related literature (Sheikhzadeh et al., 2009; Karahan & Bayraktar, 2004; Choobineh, Movahed, Tabatabaie, & Kumashiro, 2010). Also, to develop the observational checklist, an ergonomist was observed nursing movements and body mechanisms in the operating room for a duration of one month, following which, fifteen types of movements were identified. The questionnaires were given on 10 nurses, who did not form part of any study group. A briefing session was held to give participants a chance to comment on the clarity, simplicity and readability of the items. Finally, based on their comments, minor syntax errors were corrected and any ambiguous items were clarified and edited. Content validity of the instrument items was estimated quantitatively. For this purpose, the scale was reviewed by an expert panel of twenty specialists in health education, nursing and ergonomics. The panel was asked to judge the necessity and relevance of the items in order to calculate the content validity ratio (CVR) and content validity index (CVI). Finally, items having CVI less than 0.80 or CVR less than 0.62 were deleted from the scale. Internal consistency and reliability of the TTM sub-scales were estimated using Cronbach’s α and the test-retest correlation coefficients, and an estimate of α≥0.70 and correlation coefficient ≥0.61 were considered an acceptable value (Dehdari, Rahimi, Aryaeian, Gohari, & Esfeh, 2013); the inter-rater reliability was measured for the checklist, simultaneously by the two ergonomists, who then independently filled in the checklist. Kappa values ≥0.6 were considered satisfactory (Nathan et al., 2015). Both groups completed the questionnaires prior to intervention. The checklist was completed by observers before the intervention. Based on pre-test results of both groups, an educational intervention was developed for nurses in the intervention group, while the control group received no intervention. Finally, the two groups were followed up one and six months after the intervention.

#### 2.2.1 Instruments for Assessing TTM Variables

2.2.1.1 Decisional Balance Scale

Eight items described the perceived pros for good body posture (e.g. ‘If I maintain correct body posture, I can prevent low back pain); five items described the perceived cons (e.g. ‘I would have less time for my work if I adopt correct body posture’). These items were measured on a Likert scale ranging from 1=‘completely disagree’ to 5=‘completely agree’.

2.2.1.2 Self-Efficacy Scale

Six items were used to measure self-efficacy (e.g. ‘Even if I am tired, I can adopt correct body posture in the operating room’); these items were measured on a Likert scale ranging from 1 =‘completely unconfident’ to 5 =‘completely confident’.

2.2.1.3 TTM Processes of Change Scale

In this study, four experimental and three behavioral processes were used to move nurses in the intervention group into the action stage. The experimental processes of change scale was an 11-item measure with four sub-scales including: 1) awareness-raising (three items; e.g. ‘I always search for instructional materials that can enhance my awareness regarding correct body posture’); 2) environmental reevaluation (two items; e.g. ‘If my colleagues and I maintain correct posture, our work place will be safe’); 3) self-reevaluation (three items; e.g. ‘I think if I do not observe correct posture, how would my fitness be in the future’) and 4) social liberation (three items; e.g. ‘there are facilities in operating room that nurses can use for adopting correct body posture’). All the above items were measured on a Likert scale ranging from 1=‘never’ to 5= ‘very often’.

The behavioral processes of change scale was a 7-item measure with three sub-scales including: 1) self–liberation (two items; e.g. ‘I promise myself to consider adopting correct body posture as part of my occupational life’); 2) reinforcement management (two items; e.g. ‘I praise myself when adopting correct body posture’) and 3) counter conditioning (three items; e.g. ‘in my shifts, I can do stretching moves instead of wasting time’). These items were measured on a Likert scale ranging from 1=‘never’ to 5=‘very often’. Higher scores on these two scales indicate that participants use the experimental and behavioral processes of change more frequently.

2.2.1.4 Staging Algorithm for Adopting Correct Body Posture

Stage of change for adopting correct body posture was measured by a 5-item algorithm, a short staging algorithm, which is useful and valid for various behaviors (Prochaska & Velicer, 1997). According to this algorithm, nurses were categorized into one of the five stages of readiness to change based on the following answers to this question: “Do you engage in behaviors for correct body posture in the operating room?” 1) No, and I do not intend to adopt correct body posture in the next 6 months (pre-contemplation); 2) No, but I intend to adopt correct body posture within the next 6 months (contemplation); 3) No, but I intend to adopt correct body posture within the next 30 days (preparation); 4) Yes, I have adopted a correct body posture, but for 6 months or less (action); 5) Yes, I have adopted a correct body posture for more than 6 months (maintenance).

#### 2.2.2 Observational Checklist for Body Posture Practices

Fifteen items were used to measure work-related body posture in the operating room, including: standing, bending, twisting, body mechanics and body posture while handling trays and instruments, lifting, pulling, pushing, posture during work (e.g. remaining in wanted posture during a long surgery), using comfortable chairs when doing nursing procedures, wearing suitable shoes during work, extending, carrying, lifting patients, transferring patients and positioning a patient. The checklist developed included items such as movements (Yes/No/Not Observed), and was completed by the observers at baseline and 1 month and 6 months after the intervention.

### 2.3 Developed Educational Intervention

The instructional intervention was designed based on the analysis of the findings of the two groups’ pre-tests, which showed that only half of the nurses agreed with the advantages of adopting correct body posture, advantages such as preventing body spasms, improved flexibility of the body and the joints, relaxed sleeping at night and fitness. A low percentage of the nurses believed that they could correctly manage body posture despite boredom, stress and the busy operating room. Based on the nurses’ opinions the main barriers for adopting correct body posture included their shyness, the lack of adequate and safe equipments (e.g. suitable seat to sit during intervals between surgeries or non-standard trolleys), being arrogant and putting themselves before their work, instead of doing their job; only half of the nurses said that they sought educational sources to become aware of adopting correct body posture and only a small percentage contemplated on the pros and cons of correct body posture. With regard to environmental reevaluation, a limited percentage of the nurses believed that adopting correct body posture would improve the quality of task performance in the operating room, making it a healthy working environment; moreover, a low percentage of the nurses reported that they felt good about adopting correct body posture and most of the time they wondered whether their fitness in the future would be affected if they did not observe correct body posture (self-reevaluation). Most of the nurses reported that there were insufficient sources of information/data on adopting correct body posture in the operating room and that there are many nurses, who do not maintain correct body posture (social liberation). Less than half of the nurses praised themselves after adopting correct body posture and encouraged themselves for adopting it. About half of them expected that following adopting body posture they should be praised by others (reinforcement management). A small percentage of the nurses stated that they availed of every opportunity to rest during the operation, by doing movements and deep breathing (counter conditioning). A small group of nurses said that they mostly told themselves that they could observe correct body posture and make it as part of their work routine (self-liberation).

The ergonomics educational intervention involved four 60-minute sessions implemented during a four week period for the nurses in the intervention group and one instructional session for the head nurses. During the educational sessions, the nurses were divided into small groups and received appropriate instructions for adopting correct body posture through lectures, group discussions and question and answer sessions. Moreover, before the instructional sessions for the nurses, in a session with the head nurses, the importance of having a more appropriate operating room environment regarding positioning of the instruments (for the effects on the posture of nurses using the instruments) was emphasized; during this session with the head nurses, they were encouraged to contact and coordinate with the hospital managers to provide up-to-date equipment and discard timeworn instruments which could negatively affect the body posture of the nurses in the intervention group. Finally, it was decided to encourage those nurses who tried more and made more effort to adopt correct body posture. The first training session (for the nurses) was an introduction to the importance of occupational low back pain, the most important risk factors, the importance of adopting correct body posture in the operating room and its advantages were explained; at the end of this session, an instructional pamphlet on the issue was given to the nurses. In the second session, using a multi-media CD which included several clips/movies about body posture; correct body posture in the operating room was taught to the nurses and they were encouraged to learn and adopt appropriate correct posture in the operating room. In the third session, by posing some open-ended questions, the nurses were asked to relate/express their experiences and positive or negative feelings upon adopting correct body posture and its perceived advantages/disadvantages. They were asked to imagine themselves after adopting the correct body posture and tell the group members about it. In this session, the nurses were assured that they could adopt correct body posture in the operating room. In addition, they were asked to develop commitment to adopting and maintaining correct body posture in the operating room, in a way that it becomes a habit for them. They were also instructed to encourage other nurses to adopt correct body posture in the operating room. Moreover, three posters illustrating the dangers of incorrect body posture and related slogans were hung in the operating room. In the fourth session, besides nurses discussing the positive experiences of correct body posture, they were also instructed on ways of reducing stress and trying to avoid rushing through their tasks; they were also trained in doing stretching movements during any their free time in the operating room; an audio CD about gradual body relaxation was distributed among the nurses as well.

### 2.4 Data Analysis

Data were analyzed using the SPSS statistical software package (SPSS Inc., Chicago, IL, USA). The homogeneity of baseline data in demographic characteristics, TTM variables and body posture of the two groups were analyzed by χ^2^ and independent-samples *t* tests. Normality of the data was also examined and confirmed by the Kolmogorov–Smirnov test. Differences between the baseline and the one month assessments and those documented six months after the intervention in each group were tested using one-way repeated measurement analysis. Differences in TTM variables (except for the stages of change) and body posture between the groups were also tested using mixed model repeated measurement. Whitney *U*-test was used to identify significant differences in stages of change between the groups and Friedman test to identify significant differences in stages of change in each group before and one and six months after the intervention; p values<0.05 were considered significant.

## 3. Results

### 3.1 Reliability of the Sub-Scales of Developed Instrument

The correlation coefficients, Cronbach’s alpha and Kappa values of the sub-scales of developed instrument were presented in Table 1.

**Table 1:** The Cronbach’s Alpha, test–retest correlation coefficient and kappa values of the sub-scales of the instrument

Sub-scales	Cronbach’s Alpha	Test–retest correlation coefficient	Kappa values
Perceived pros	0.85		
Perceived cons	0.86		
Self-efficacy	0.78		
Awareness-raising	0.88		
Self-reevaluation	0.84		
Environmental reevaluation	0.85		
Social liberation	0.80		
Self–liberation	0.80		
Reinforcement management	0.99		
Counter conditioning	0.78		
Stage of change		P=0.01, r=0.81	
Adopting correct body posture			0.80

### 3.2 Demographic Characteristics of the Participants

The mean age (SD) of the nurses in the intervention (70% women) and control (62.5% women) groups was 31 (5.8) and 32 (6.7) years respectively. Nurses in the intervention and control groups had an average of 2.7 (SD = 7) and 2.9 (SD = 6.6) years of working experience in the operating room, respectively. Also, prior to the implementation of interventions, no significant differences were found between the any of the demographic characteristics of the two groups (p<.05).

### 3.3 Individuals’ Readiness Stages for Change at Baseline and Follow-Up

As shown in Table 2, at baseline, all of the nurses in both groups were in the contemplation and preparation stages. Results showed that after the intervention, significantly more nurses in the intervention group moved into the action stage (61.9% at post-test 1 and 50% at post-test 2) compared to the control group (0% at post-test 1 and 10% at post-test 2) (p<0.05); however significantly fewer nurses in the intervention group were in the contemplation and preparation stages following the intervention (see Table 2).

**Table 2:** Distribution of nurses according to TTM stages of readiness to change for adopting correct body posture in the before and one-month and six-months after educational intervention in the intervention (n=42) and the control group (n=40)

	Pre-intervention		One-month after intervention		Six-months after intervention	

	Intervention group	Control group	Intervention group	Control group	Intervention group	Control group
Precontemplation	0 (0.0)	0 (0.0)	0 (0.0)	1 (2.5)	0 (0.0)	3 (7.5)
Contemplation	19 (45.2)	21 (52.5)	1 (2.4)	24 (60)	1 (2.4)	27 (67.5)
preparation	23 (54.8)	19 (47.5)	15 (35.7)	15 (37.5)	20 (47.6)	6 (15)
Action	(0.0) 0	0 (0.0)	26 (61.9) *^†^	0 (0.0)	21 (50) *^†^	4 (10)
Maintenance	0 (0.0)	0 (0.0)	0 (0.0)	0 (0.0)	0 (0.0)	0 (0.0)

1. Result of Mann Whitney U-test;

* P < 0.05 compared to the control group;

2. Result of Friedman test;

† P < 0.05 compared to pre-intervention values;

3. +Stages with 0 cells did not include in the analysis;

### 3.4 Comparison of Differences in TTM Variables and Adopting Correct Body Posture at Baseline and Follow-Up

No significant differences were found between the two groups for any TTM variables and adopting correct body posture (p<0.05) before the interventions. As shown in Table 3, results showed that following the intervention, the intervention group had higher total mean scores on self-efficacy, pros of correct body posture, self-reevaluation, environmental reevaluation, social liberation, experimental processes, self–liberation and correct body posture (p<0.05) compared with the control group. In addition, one and six months after the intervention, no significant differences (p<0.05) were found for the perceived cons, awareness-raising, reinforcement management, counter conditioning and total scores of behavioral processes between the two groups (see Table 3).

**Table 3:** Comparison of TM variables and adopting correct body posture before and one-month and six-months after educational intervention in the intervention (n=42) and the control group (n=40)

	Pre-intervention		One-month after intervention		Six-months after intervention	

	Intervention group	Control group	Intervention group	Control group	Intervention group	Control group
Pros of correct body posture	33.55±7.27	32.25±3.63	34.02±3.19	31.73±3.50	34.26±3.11^†^	31.20±3.09
Cons of correct body posture	24.19±3.83	23.32±4.34	22±3.97	23.75±4.03	21.48±3.91*	23.60±3.84
Self-efficacy	12.62±4.51	11.88±4.82	15.26±4.13	11.80±4.61	14.74±3.88*^†^	11.93±4.71
Awareness-raising	8.83±2.46	8.98±2.54	9.74±2.39	10.08±2.18	9.79±2.50*	10.18±2.09
Environmental reevaluation	6.74±2.20	7.28±1.81	7.21±1.94	7.18±1.68	7.48±1.84*^†^	7.28±1.74
Self-reevaluation	10.79±2	10.58±2.14	11.40±1.84	10.45±2.06	11.48±1.49*^†^	10.68±1.72
Social liberation	7.52±1.78	7.15±1.80	8.40±1.68	7.08±1.79	8.83±1.65*^†^	7.23±1.48
Total score of experimental processes	33.88±5.16	33.97±4.58	36.76±4.80	34.78±3.51	33.98±4.93*^†^	34.90±3.63
Counter-conditioning	7.74±2.21	7.33±1.80	8.50±2.13	7.30±1.79	8.67±1.88*^†^	7.68±1.65
Reinforcement management	5.48±1.56	5.40±1.74	6.02±1.40	5.33±1.61	6.31±1.11*	5.70±1.38
Self-liberation	6.69±1.44	6.23±1.98	7.31±1.39	6.08±1.77	7.21±1.44*^†^	6.08±1.54
Total score of behavioral processes	19.90±3.30	18.95±3.61	21.83±3.10	18.70±3.38	21.76±2.94*	18.75±3.45
Adopting correct body posture	19.5 ±3.81	18.2±4	25.41±4	19.45±4.15	30.33±4.02*^†^	19.7±4.21

1. Values are Mean ± SD

2. Result of one-way repeated measurement analysis;

* P< 0.05 compared to pre intervention.

3. Result of mixed model repeated measurement;

† P < 0.05 compared to the control

## 4. Discussion

Results of the present study showed that one month and six months after the intervention, considerable progress occurred in terms of nurses’ stages of change in the intervention group, compared to the controls. Based on TTM, adopting correct body posture score should increase accordingly as individuals’ progress through the stages. The findings of this study indicate that the mean score of adopting correct body posture score among nurses in the intervention group could be improved through the educational program conducted. This finding was consistent with Munchaona (2003) and Woods et al. (2002). In agreement with findings of similar studies (Molaison & Yadrick, 2003; Kim & Cardinal, 2009; Shirazi et al., 2007), results of the present study showed that stage-matched educational intervention, could move the individuals to later stages of readiness to change.

In addition, results showed that the mean score of self-efficacy among nurses in the intervention group increased significantly following the intervention. The findings were also consistent with those of similar studies (Gorely & Bruce, 2000; Lubans & Sylva, 2006; Sol, Graaf, Petersen, & Visseren, 2011). Progressively moving individuals into the later stages of readiness to change, increases their self-efficacy, as reported by Keller, et al. (2001), who showed that the self-efficacy scores of a German administration unit with regard to adopting correct body posture differed in the five different stages of change and those who were in later stages of change were found to be more self-confident (Keller et al., 2001). In our study, more nurses in the intervention group advanced to the action stage, demonstrating a significant difference between the groups following the intervention. Given the importance of self-efficacy to adopt correct body posture among nurses, researchers should focus on developing ergonomics-oriented educational interventions aimed at increasing self-efficacy among them.

We also found that the intervention considerably increased perceived pros in the intervention group compared to the control group 1 month and 6 months following the intervention. Mohammadi Zeidi et al. (2011) showed that developing a TTM-based ergonomics educational program can considerably enhance the advantages of adopting good body posture among computer users (Mohammadi Zeidi et al., 2011). Numerous studies have also shown that individuals in the later stages of change had more perceived pros than those in the earlier stages (Shirazi et al., 2007; Sarkin, Johnson, Prochaska, & Prochaska, 2001). Both the above-mentioned data and our findings affirm that by designing ergonomics educational interventions to progressively move nurses into the later stages of change (e.g. action and maintenance), the advantages of adopting correct body posture can be appropriately instructed.

While the results of this study indicate the effectiveness of the educational intervention on the perceived pros of correct body posture, the mean scores of cons among the nurses in the intervention group, did not show any significant decrease compared to the control group, findings that are consistent with TTM assumptions. The balance between benefits (pros) and costs (cons) of changing a health behavior for progression within stages is important. Participants whose perceived pros outweigh their perceived cons are more motivated to adopt recommended behavior (Prochaska & Velicer, 1997). In this study, the pros of correct body posture outweighed the cons before and following the intervention among the nurses; this finding was supported with Gorely and Bruce (2000), but was contradicted with Mohammadi Zeidi et al. (2011), who reported that educational interventions could have significantly decreased the perceived cons of computer users for adopting correct body posture, a paradox, which may be explained by the sample of the study (Mohammadi Zeidi et al., 2011). Since nurses are considered healthcare staff, it goes without saying that compared with other non-health care occupations, they are more aware of the advantages of the correct body posture.

Overall, the present study showed that while there was no significant difference in behavioral processes of change (except for self-liberation) between the two groups. Total mean scores of all experimental processes (including environmental reevaluation, self-reevaluation, social liberation and counter conditioning variables) were higher in the intervention group than the controls following the intervention. In other words, use of these processes of change caused the nurses to advance into the action stage, which was supported with Kirk et al. (2010) (Kirk, MacMillan, & Webster, 2010). The TTM assumes that the behavioral processes are most often used in the later stages (i.e. maintenance and action) whereas the cognitive processes are most often used in the early stages of change (i.e. pre-contemplation, contemplation and preparation) (Prochaska & Marcus, 1994). Since nurses in the current study were in the contemplation and preparation stages, they mainly used the experimental processes of change for moving through the earlier stages.

### 4.1 Implication for Future Research

Despite high prevalence of low back pain among operating room nurses and the importance of ergonomics education to reducing their WMSD, to the best of our knowledge; no educational intervention has been developed on this issue. Current educational programs regarding this issue for nurse will never be sufficient. Further theory-based education efforts should be established for operating room staff about measures that should be taken to prevent low back pain in-service training. It is recommended that further similar researches be designed and conducted for nurses in other hospital wards.

## 5. Conclusion

Results of the present study showed that the educational ergonomics intervention based on TTM can progressively facilitate significant change in movement of nurses from the contemplation and preparation stages to the action stage of adopting correct body posture in the operating room.
